# Testing of a reusable chemical warming pad and an insulating jacket to manage hypothermia of preterm or low birthweight neonates

**DOI:** 10.1038/s41598-025-96275-1

**Published:** 2025-04-10

**Authors:** Anisuddin Ahmed, Fariya Rahman, Md. Mahinur Islam, Mohammad Hridoy Patwary, K. M. Tanvir, Saifuddin Ahmed, Abul Hussam, Md. Mominul Islam, Mats Målqvist, Ahmed Ehsanur Rahman, Shams El Arifeen, Syed Moshfiqur Rahman

**Affiliations:** 1 Global Health and Migration Unit, Department of Women’s and Children’s Health, Uppsala University, Akademiska sjukhuset, 751 85 Uppsala, Sweden; 2 Maternal and Child Health Division, International Centre for Diarrhoeal Disease Research, Dhaka, Bangladesh; 3 Department of Chemistry and Biochemistry, The Florida State University, Tallahassee, USA; 4 Institute of Statistical Research and Training, University of Dhaka, Dhaka, Bangladesh; 5 Department of Population, Family and Reproductive Health, Bloomberg School of Public Health, Johns Hopkins University, Baltimore, USA; 6 Center for Clean Water and Sustainable Technologies, Department of Chemistry and Biochemistry, George Manson University, Fairfax, USA; 7 Department of Chemistry, University of Dhaka, Dhaka, Bangladesh; 8 Centre for Health and Sustainability, Department of Women’s and Children’s Health, Uppsala University, Uppsala, Sweden

**Keywords:** Sodium Acetate Trihydrate, Paraffin, Glycerol, Hypothermia management, Thermoregulatory device, Thermal Jacket, Kangaroo Mother Care, Medical research, Chemistry, Materials science

## Abstract

Hypothermia remains a leading contributing factor to neonatal mortality. This study reports testing of a thermoregulatory device—‘Thermal Jacket’ that includes a reusable chemical warming pad (CWP) and an insulating jacket designed for hypothermia management. The laboratory experiments were conducted in two distinct phases between February’21 and June’22. In phase 1, a ternary composite of Sodium-Acetate-Trihydrate, Glycerol, Paraffin, and water contained in a high-density polyethylene-pouch named ‘CWP’ was finalised, and an insulating jacket was designed for targeted heat retention. In phase 2, the device’s efficacy was evaluated using a mannequin in a controlled setting. The sample size was 81 events. Welch’s t-test, ANOVA, and GEE were used to assess any significant differences between successful and failed events. Among 81 events, approximately 93% events of CWP and 98% events of insulating jacket successfully maintained temperature within 36–38°C for 120 minutes. Moreover, ambient temperature, reuse of CWPs, humidity did not have any significant effect on the success rate of the CWP and insulating jacket. Thermal Jacket had achieved and sustained the temperature range of 36–38°C for 2 hours. While this study used mannequin, clinical trial with preterm or low birthweight neonates is imperative to assess its effectiveness for thermal care management.

**Supplementary Information:**

The online version contains supplementary material available at 10.1038/s41598-025-96275-1.

## Introduction

Premature delivery with low birthweight (LBW) (birthweight less than 2500 g)^1^ is a serious global concern. Annually, over 15 million neonates are born preterm (<37 completed weeks of gestation)^2^ and an estimated 20 million neonates are born with LBW worldwide^1^. These vulnerable neonates account for global neonatal deaths with approximately 16%^3^. In Bangladesh, the preterm birth rate is 16.2% and LBW rate is 23%^4^.

The World Health Organization (WHO) recommends Kangaroo Mother Care (KMC) for managing preterm and LBW neonates in which a mother or a caregiver has to provide skin-to-skin care for maintaining euthermic condition (36.5–37.5°C/97.7–99.5°F-body temperature) of a neonate for 8–24 hours per day^5^. KMC has been proven to be a safe, efficacious, and cost-effective intervention in different health facility settings^6,7^. However, there are gaps that remain in maintaining continuous skin-to-skin care practices^8–13^. Mothers of KMC-eligible neonates are convinced and willing to provide KMC for their offsprings’ wellbeing, but lack of support from family members for the mothers’ personal and resting time during the hospital stay and pain/fatigue of mothers (e.g. immediately after caesarean section, other illnesses) negatively affect the skin-to-skin care practice^14,15^. Moreover, though the transportation in KMC position is beneficial^16^, providing KMC during emergency transportation and referral from home and one healthcare facility to another imposes a great challenge^17^.

Various thermoregulatory devices have been used to supplement KMC. Though all have reported to be efficacious to maintain euthermia of the sample population, it is imperative to cast an analytic lens to find the opportunities of improvement. For example, a few of the devices come with only a heat pad and lack any insulating swaddle or jacket^18,19^. Another device cost as high as USD 190.0 to 200.0^20,21^ which may remain unaffordable in low-resource setting’s context.

To address all these challenges, we developed a thermoregulatory device named ‘Thermal Jacket’ for maintaining euthermic temperature of preterm and LBW neonates in conjunction with the recommended KMC practice, especially when providing KMC is not feasible. Our aim is to use this device as a supplement for skin-to-skin care practice for a caregiver (especially mother) and the premature or LBW neonates during hospital stay, referral, and/or emergency transportation, in between KMC sessions and while KMC provider rests. Consequently, by the use of the device, we aim to increase KMC uptake in Bangladesh and similar low and middle income countries.

The thermal jacket includes two reusable components which are a chemical warming pad (CWP) and an insulating jacket. In this paper, we aim to report the design and development of the CWP and the insulating jacket and assess their performance in a controlled laboratory setting to check if the CWP and the insulating jacket can attain and maintain a temperature range between 36˚C and 38˚C for 120 mintues.

## Materials and methods

**Fig. 1:**
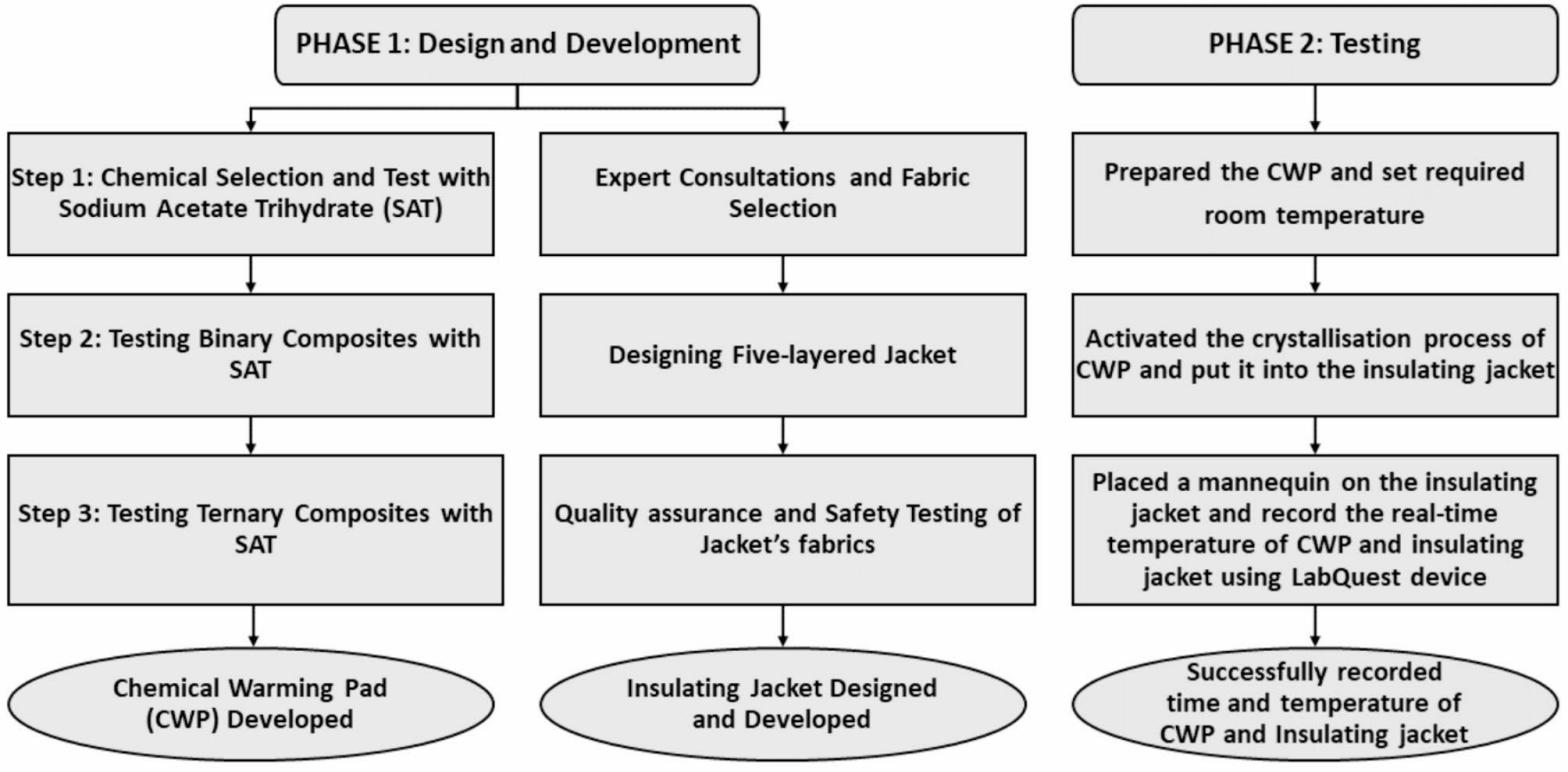
Design, development, and testing phases of the chemical warming pad and the insulating jacket.

We conducted the laboratory testing between February 2021 and June 2022 to design and develop the thermal jacket and optimise its performance. The thermal jacket design, development, and testing experiments were conducted in two phases (Fig. 1). In phase 1, we adopted the trial and error method to fix a chemical composition used for the chemical warming pad. Once the composition was finalised, we decided its packaging. Simultaneously, we designed an insulating jacket with appropriate fabrics considering sufficient insulating capacity to ensure heat retention. In phase 2, we tested the capacity of the CWP and the insulating jacket to attain and maintain temperature between 36–38°C. In this phase, we trialed the thermal jacket on a mannequin in a controlled laboratory environment subsequently. The LabQuest devices^22^ were used to record the retention time and temperature starting from the crystallisation of the solution inside the CWP.

We tailored the insulating jacket through consulting designers and a textile manufacturer, Poeticgem International Ltd. Furthermore, the quality of fabrics of the insulating jacket had been checked by Intertek Textile Ltd. in Bangladesh. All the laboratory testings of the CWPs and the insulating jackets were carried out in icddr, b and Material Chemistry Research Laboratory under the Department of Chemistry of the University of Dhaka.

### Phase 1

#### Design and development of chemical warming pad (CWP)

In step 1, we chose a phase-change material, Sodium Acetate Trihydrate (SAT)^23^ for the CWP as the primary source of heat generation due to its high latent heat of fusion (250 kJ/kg) (energy released or a thermodynamic system during a constant-temperature process), extensive cooling range, non-hazardous property, availability and low-cost^23–25^. For thermoregulatory devices, SAT is vastly used for long-term thermal energy storage in a metastable supercooled liquid state^24^ at low ambient temperature. It releases heat on demand by initiating the crystallisation^25^ using metal triggers in the aqueous solution. Despite such thermo-physical properties of SAT, there are a few challenges associated, including hard, lumped, big, and sharp edges of SAT crystallites, unintentional nucleation in a metastable supercool liquid state, and fast crystal growth during solidification which causes low retention time for our desired temperature^26–28^. Moreover, when the number of thermal cycles increases, the latent heat capacity of SAT also decreases^25^. This poor thermal cyclic stability limits its capacity to maintain our desired temperature range.

In step 2, to overcome the limitation of the aquous SAT, we experimented on a few binary composites to land up on the solution with improved thermal cyclic stability. In the experiments, Graphite^29^, Carboxyl Methyl Cellulose^30^, Sodium Chloride^31^, Glycerol^31^, Ethylene Glycol^32^, and Paraffin^33^ were used as additives with the aqueous SAT solution but none could meet the study objective, as uncontrolled nucleation, low heat retention, and attainment of high temperature than desired were observed (Supplementary Table 1).

In step 3, guided by the evidence, glycerol and paraffin compositions had the potentials to achive the desired temperature range and heat retention duration. Therefore, we tested a ternary composite of Glycerol and liquid Paraffin with an aqueous SAT solution through a eutectic system (a homogenous mixture of materials that melts or solidifies at a single temperature)^34^ approach to obtain the desired latent heat capacity with desired temperature and retention time finally. As the crystallisation temperature and heat retention, both are high for the SAT aqueous solution and binary combination with paraffin, we used varying amounts of glycerol to the binary mixture and tested its heat retention duration in the desired temperature (36–38°C) range. Various mass fractions of glycerol (5%, 15%, 20%, 27%, and 35%) mixed with the binary solution were tried out while crystallisation temperature with retention time was observed.

#### Design and development of insulating jacket

The selection of the jacket fabrics, and design followed a meticulous process. Expert consultations with apperal designer, safety and quality check officials of the ready-made garments (RMG) industry, public health researchers, and neonatologists were taken place in several rounds, to select the most suitable and comfortable fabrics for the jacket. Moreover, safety, availability, and affordability of the fabrics were also considered during the selection and design of the insultaing jacket.

Through iterative process, we designed a five-layered insulating jacket comprising single jersey, polyester-polar fleece, Taffeta, screemline padding, and water-resistant polyester, from inside to outside and polyester ripstop for the kangaroo pocket to keep the CWP inside the jacket (Supplementary Fig. 1). These fabrics have a high insulating capacity and low-to-moderate air permeability that protect against loss of heat caused by convection, conduction, evaporation and radiation (Supplementary Table 2). Moreover, the fabrics are lightweight, and the outer layer and the fabric of the kangaroo pocket are water-resistant.

The fabric of kangaroo pocket that contains the CWP does not hold any generated heat to itself and therefore, the neonates put inside the jacket will receive the total heat generated by the CWP. The length of the pocket covers the maximum body surface area of the neonate from neck to buttock. Again, the inner body touching layer of the jacket is jersey cotton, followed by fleece, wadding wrapped in taffeta and the outer layer is waterproof polyester. Each fabric serves distinct purpose of the jacket. For example, cotton provides softness, fleece helps to keep the warmth inside by creating an insulating environment. Similarly, the third layer bolsters the first two layers by providing more comfort and restriction of heat transport. Finally, the outermost layer prohibits air and water passing from outside and thus ensures overall protection for the baby. We used soft Velcro on the side and bottom edges of the jacket to make sure the jacket does not get unwrapped during movement. Moreover, the jacket is designed in such a way that it covers a neonate from head to toe given that except from the body cover, it also has a hoodie to support warmth conservation of the head surface area.

### Phase 2

#### Laboratory testing of thermal jacket (CWP and insulating jacket)

The CWP and insulating jacket together were tested on a mannequin in a controlled laboratory environment at icddr, b.

##### Sample size Estimation for laboratory testing of the thermal jacket

Our objectives were to determine whether the thermal jacket attains and maintains a temperature ranging between 36 and 38°C for 120 minutes and the effect of ambient temperature on the thermal jacket’s performance. Moreover, we were also interested in determining the effect of the reusability of a CWP on the insulating jackets’ performance. For this, we calculated the required number of events for capturing a 95% success rate for the CWPs as well as the insulating jackets using the below formula:

$$\:n=\:\frac{{Z}^{2}P(1-P)}{{d}^{2}}\:$$

Considering,

$\:Z$ is the Z-score corresponding to 95% confidence level = 1.96, $\:\:d$ is the margin of error = 0.05, $\:P$ is the rate of success = 95%, the calculated sample size was 73 events. The definition of an event was to put the mannequin inside the thermal jacket and observe the temperature of the CWP and insulating jacket simultaneously for 120 minutes. Since, the events were correlated within the CWP, multiplying the sample size by a design effect of 1.08 considering the intra-cluster correlation coefficient 0.01, the minimum required events became 73 × 1.08 $\:\approx\:$ 79. Since each CWP was planned to reuse nine times due to its functional performance limit to maintain the temperature range 36–38⁰C for 120 minutes, we needed nine CWPs to observe the required number of events. Hence, the CWPs and insulating jacket were tested 81 times with a mannequin to assess their performance to attain and maintain the desired temperature range (36–38⁰C) for 2 hours.

##### Sampling technique for the laboratory testing of the thermal jacket

For assessing the effect of ambient temperature on the performance of the CWP and the insulating jacket, we artificially created room temperature ranging from 18–34⁰C. Three temperature bands were created within the range, including 18–24⁰C, 25–29⁰C, and 30–34 ⁰C. Furthermore, we tested 81 sample events in each temperature band more than once. The detailed description is provided in the supplementary Table 3.

##### Temperature measurement of the CWP and insulating jacket

**Fig. 2:**
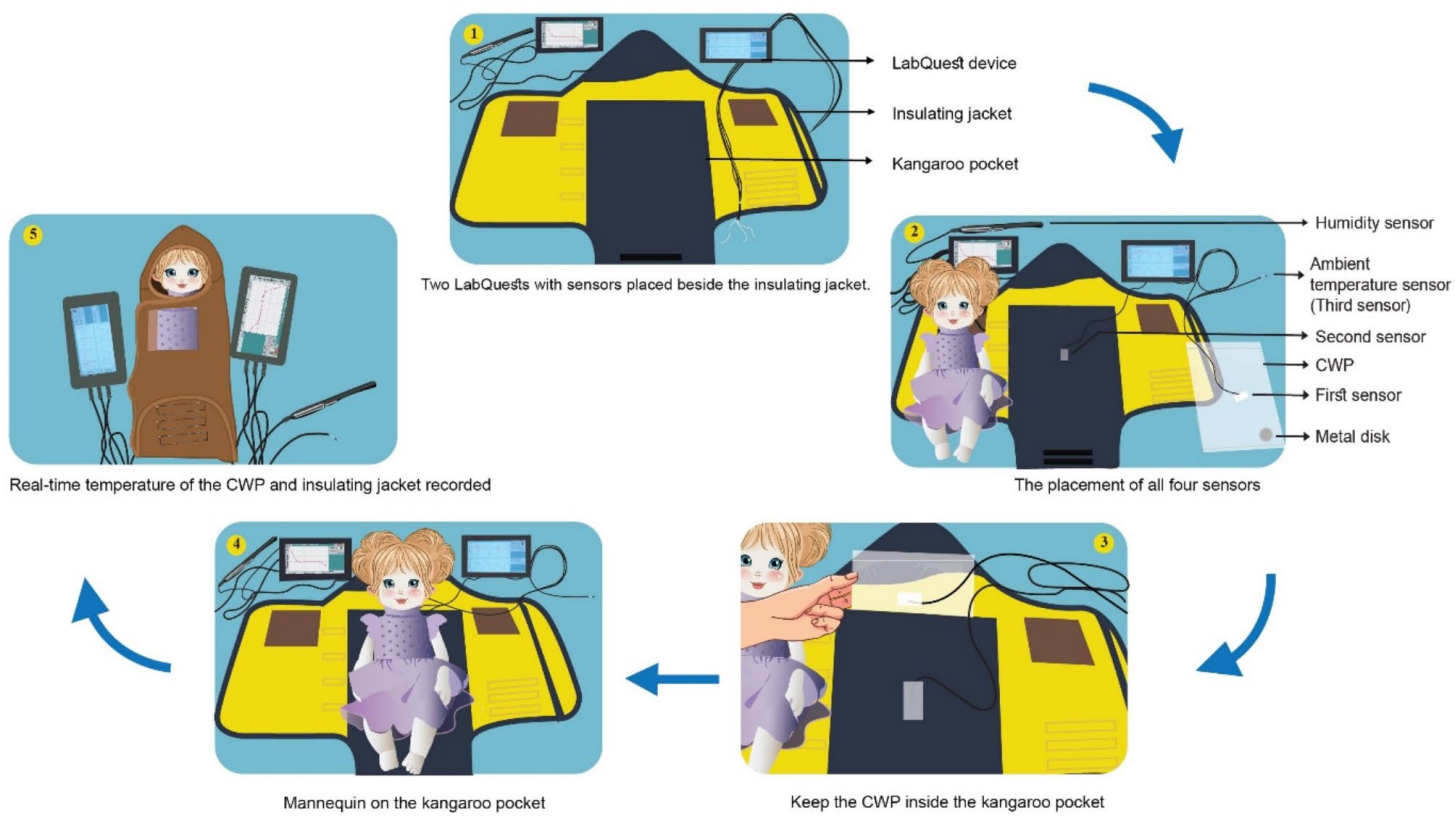
Temperature measurement process of the CWP and insulating jacket.

To test the thermal jacket in the laboratory, we have devised LabQuest to measure the real-time temperature of different surfaces of the CWP and insulating jacket (Fig. 2). The first sensor was attached to the surface of the CWP, the second one was on the surface of kangaroo pocket of the insulating jacket, and the third one was kept outside of the jacket to measure ambient temperature. One additional sensor was also used to measure the humidity of the room, separately. Once all the sensors were placed correctly, we then flexed the metal trigger inside of the CWP and observed the initiation of crystalisation. Once the liquid starts to solidify, we then put the CWP in the kangaroo pocket of the insulating jacket instantly. A mannequin was then placed on the kangaroo pocket immediately and wrapped inside the the jacket.

Once the jacket is prepared, we started recording the time to measure the temperature and humidity. The data points were collected on an interval of every 6 seconds. Therefore, for every one minute, we got 10 data points. Though, our objective was to observe if the thermal jacket can attain and maintain the temperature range between 36⁰C and 38⁰C for 120 minutes, but we recorded the performance for 130 minutes, so that we could observe if the jacket has potential to perform beyond 120 minutes and give caregivers enough time to put the neonates off the jacket in a real-life setting. We performed all the events in the same manner.

##### Data analysis

We used the data analysis software ‘R’, version 4.4.1 (https://www.r-project.org/)^35^ to compute the result. The temperature was measured in degree celsius and the time was in minutes. The simple mean and standard deviation had been estimated both for the CWP and insulating jacket, seperately. The Welch’s t-test had also been performed to see any potential differences in the performances between the CWPs and the insulating jacket. Moreover, these performances had been assessed by ambient temperature, humidity, number of times CWP were reused, and number of CWPs were used with simple mean and standard deviation. Analysis of Variance (ANOVA) had also been performed to test whether there is any statistical differences between the mentioned factors.

An event had been labelled as successful if it attained a temperature of 36°C within three minutes (maximum) from the baseline point and then maintained a temperature range between 36°C and 38°C for 120 minutes. If the temperature exceeded or dropped from the above range at any time point and returned to the range with a rate of 0.5°C/hour or more, we considered it as a successful event. The percentage of events that successfully attained and maintained the temperature range 36–38⁰C for 120 minutes was then calculated and the success rate was disaggregated by ambient temperature, number of times CWP were reused, and number of CWPs were used to see whether there was any significant association between the success status of an event and these factors.

To estimate the adjusted effect of the above factors on the success status of an event, an extended version of the generalized estimating equation (GEE) known as ‘geefirth’ model was performed^36^. Since each of the CWPs was reused several times, the panel data-based method ‘geefirth’ model was then used to adjust correlation within the CWPs while estimating the effect of ambient temperature bands, average humidity, and number of times CWP were reused on the success of an event. This model also reduces bias due to the small sample size, as well as resolves the separation issue (that occurs when covariates perfectly [or near perfectly] predict the outcome) arising from the skewed distribution of the success rate of the events^36^.

## Results

### Determination of optimum mass fraction of glycerol and paraffin in SAT aqueous solution

**Fig. 3:**
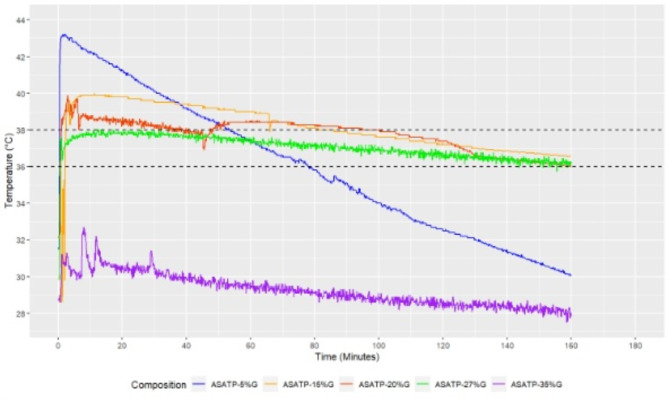
Time-dependent freezing curves of ASATP-5%G, ASATP-15%G, ASATP-20%G, ASATP-27%G, ASATP-35%G.

We have found the optimised composition- aqueous solution of SAT + Paraffin + 27%Glycerol (ASATP-27%G) that maintained the temperature between 36°C and 38°C for more than 120 minutes (Fig. 3). The ASATP composite treated with 27% Glycerol showed an increase in heat retention duration at the required temperature range without substantially altering the other thermophysical parameters.

### Performance measurement of chemical warming pad and insulating jacket

The temperature of the CWP seemed to be higher than the temperature of the insulating jacket in all instances within 120 minutes shown in Table 1. The maximum mean temperatures reached by the CWPs or the insulating jacket were less than 38⁰C and there were slightly statistically significant differences in temperature between the CWP and jacket. The CWP took significantly less time to reach the euthermic band compared to the jacket (0.95 min vs. 1.18 min). Both the CWP and insulating jacket increased their temperature by more than 6⁰C per minute to reach the euthermic band when the metal trigger was flexed. As the CWP is a heat-source itself, its temperature rose quickly and reached the euthermic band faster and took less time as compared to the insulating jacket. However, these rates of reaching the euthermic range between the CWP and insulating jacket were not statistically significantly different.

**Table 1:** Performance characteristics of CWP and insulating jacket tested for 120 minutes.

Characteristics	CWP		Insulating jacket		*p*-value^†^
	Mean	SD	Mean	SD	
Baseline temperature (°C)	31.50	1.69	30.83	2.46	0.04
Maximum temperature (°C)	37.80	0.32	37.70	0.35	0.05
Endline temperature (°C)	36.43	0.39	36.40	0.39	0.51
Time to reach temperature band* (min)	0.95	0.62	1.18	0.80	0.04
Rate of reaching euthermia band from starting time (°C/min)	6.53	4.17	6.21	4.44	0.61
Rate of decreasing from maximum to end point (°C/min)	0.01	0.01	0.01	0.01	0.16

*SD * is Standard deviation.

^*^Temperature band is Temperature between 36°C and 38°C.

^†^p-value from Welch’s t-test.

There was no statistically significant effect on the baseline mean temperature of the CWP and the insulating jacket when we used the CWPs repeatedly in different ambient temperature bands (Supplementary Table 4). Similarly, no statistically significant differences were observed in the maximum mean temperature of the CWP and insulating jacket for repeated use of the CWPs at different ambient temperature bands (Supplementary Table 4). However, the time to reach at the euthermic range significantly differed ranging from 0.50 to 1.70 minutes among CWPs and insulating jacket when different CWPs were used repeatedly (Supplementary Table 5). It can be concluded that the timing of the CWP and insulating jacket reaching the desired temperature range is consistent and reliable.

Out of the 81 events, 92.6% of the events of the CWPs and 97.5% of the events of the insulating jacket maintained temperature in the euthermic range successfully. However, the proportion of events that successfully maintained euthermic temperature was not statistically significantly different (p-value: 0.15) between the CWP and insulating jacket. Moreover, the successful performance of both the CWP and the insulating jacket in terms of maintaining temperature in the euthermic range didn’t appear to be associated with the use of different CWPs repeatedly at different ambient temperature bands (Table 2). This finding suggests that the CWP and the insulating jacket can be reused multiple times without compromising its ability to maintain the desired temperature range for 120 minutes.

From both the fitted models (Tables 3 and 4), it is evident that none of the factors including ambient temperature, humidity, and the number of reuse of CWP have any significant effect on the success rate of CWP and insulating jacket for maintaining the temperature in the euthermic range.

**Table 2:** Percentage of events that maintained desired temperature range over different factors.

	CWP	*p*-value^†^	Insulating jacket	*p*-value^†^
Ambient temperature (°C)				
18–24	88.90	0.198	92.60	0.129
25–29	88.90		100.0	
30–34	100.0		100.0	
Number of times CWP used				
1	88.90	0.113	88.90	0.518
2	66.70		88.90	
3	100.0		100.0	
4	88.90		100.0	
5	100.0		100.0	
6	100.0		100.0	
7	88.90		100.0	
8	100.0		100.0	
9	100.0		100.0	
Number of CWP				
1	100.0	0.594	100.0	0.518
2	88.90		88.9.0	
3	100.0		100.0	
4	100.0		100.0	
5	88.90		100.0	
6	88.90		100.0	
7	88.90		100.0	
8	77.80		88.90	
9	100.0		100.0	

^†^p-value from chi-square test.

**Table 3:** Factors associated with the desired temperature management by the CWP.

Success rate for CWP						
	Coefficients	SE^†^	Wald	p-value	95% CI	
Number of times CWP used					Lower	Upper
1	(Ref.)					
2	− 1.82	1.77	1.06	0.31	− 5.29	1.65
3	2.28	4.81	0.23	0.64	− 7.14	11.70
4	0.21	2.17	0.01	0.92	− 4.05	4.48
5	3.24	7.74	0.47	0.50	− 6.05	12.52
6	2.18	4.78	0.21	0.65	− 7.19	11.55
7	− 0.04	2.37	0.00	0.99	− 4.68	4.59
8	2.74	5.01	0.30	0.59	− 7.07	12.56
9	0.90	4.87	0.03	0.85	− 8.65	10.45
Ambient temperature (°C)						
18–24	(Ref.)					
25–29	− 1.77	2.78	0.41	0.52	− 7.21	3.67
30–34	2.24	4.84	0.21	0.65	− 7.25	11.73
Average humidity	− 0.09	0.08	1.18	0.28	− 0.25	0.07
Intercept	8.16	6.70	1.49	0.23	− 4.96	21.28

*Wald* is Wald test statistics.

^†^*SE* Standard error.

**Table 4:** Factors associated with the desired temperature management by the insulating jacket.

Success rate for insulating jacket						
	Coefficients	SE^†^	Wald	p-value	95% CI	
Number of times CWP used					Lower	Upper
1	(Ref.)					
2	0.03	1.68	0.00	0.99	− 3.27	3.33
3	2.56	4.91	0.27	0.60	− 7.06	12.19
4	2.3	4.69	0.24	0.63	− 6.90	11.5
5	3.72	4.55	0.67	0.42	− 5.20	12.64
6	2.25	4.98	0.20	0.65	− 7.51	12.01
7	1.09	4.54	0.06	0.81	− 7.81	9.98
8	2.23	4.82	0.21	0.65	− 7.22	11.68
9	2.20	5.19	0.18	0.67	− 7.97	12.37
Ambient temperature (°C)						
18–24	(Ref.)					
25–29	1.88	3.52	0.28	0.6	− 5.03	8.79
30–34	2.73	4.54	0.36	0.55	− 6.17	11.62
Average humidity	− 0.06	0.11	0.29	0.59	− 0.28	0.16
Intercept	5.53	8.96	0.38	0.02	− 12.03	23.09

*Wald* is Wald test statistics.

^†^*SE* standard error.

For the six events that were unable to maintain temperature between 36–38⁰C, none of them had a temperature less than 36⁰C at any time point. At the time points where temperature exceeded 38⁰C, 50% of those instances had a temperature less than 38.26⁰C for CWP and 38.4⁰C for jacket (**Supplementary Table 6**). Also, in 50% of those instances, the temperature exceeded 38⁰C and stayed for less than 21 minutes for CWP and less than 32 minutes for the jacket. The average rate of returning to the euthermic range at any time point of the maximum temperature of these events was less than 0.2°C/hour.

## Discussion

The laboratory experiment suggests, the thermal jacket successfully attain and maintain temperatures within the desirable temperature range of 36–38⁰C for 120 minutes. The stability and consistency of the results suggest that the thermoregulatory device, thermal jacket can be further tested for clinical trials.

The design of the insulating jacket matches the design of a similar device which also has five layers^37^. However, the other device relies on electricity and this may limit its utilisation in resource-limit settings including Bangladesh. Our device, therefore, has a major advantage since it does not require any electricity, thus, makes it low-cost and affordable. On the other hand, there are two more thermoregulatory devices^20,21^ similar to the thermal jacket and are aimed to support KMC. However, there is a scarcity of data as there was no paper found on the findings of laboraroty trial which deemed necessary before initiating any clinical trial, according to the Food and Drug Administration (FDA)^38^.

The CWP used in the insulating jacket to provide heat and retain temperature in the range between 36⁰C and 38⁰C is a ternary composite of aqueous SAT solution, Glycerol, and liquid Paraffin. This ternary composite is a unique and novel design to enhance the thermophysical properties of the phase-change material, like SAT that provided more precise temperature retention as compared to the binary composite of SAT and ethylene glycol tested in 2017^32^. We used ‘Glycerol’ which has a low freezing point and a suitable retention time, as a cooling agent to keep the solidification temperature under range^39^ and ‘Paraffin’ as a thickening agent to make the aqueous composition reusable^33^. The lab result showed that when the Glycerol content increases, the phase transition temperature of the target aqueous composition gradually decreases. By altering the glycerol percentage in the aqueous SAT solution and Paraffin (ASATP) composite, it is possible to modify and improve the thermophysical parameters of aqueous SAT, including its solidification temperature, degree of supercooling, and heat retention/release time. Glycerol insertion into SAT-Paraffin composite made the crystallites aid in size regulation of SAT crystallites by reducing the formation of big, lumpy SAT crystallites and helps to retain temperature between 36⁰C and 38⁰C range for more than 120 minutes.

The thermal jacket, which is a combination of a CWP and an insulating jacket was able to get into the desired temperature range within an average of one minute, highlighting its rapid response time and potential clinical utilisation as a thermoregulatory device to prevent hypothermia. During the crystallisation process, it exhibited a remarkable temperature increase exceeding 6°C per minute. Hence, preterm and LBW neonates would get an external heat source with 36–38°C quickly, as per their requirement.

Notably, reusability of CWPs and ambient temperature do not have any impact on the performance of the thermal jacket, hence can be used in different ambient temperature, as required. This characteristic itself makes this innovation novel and potential to positively influence the survival of many preterm and LBW neonates.

One study reported that a neonate might get burn if it is exposed to any warming devices whose maximum temperature exceeds 42°C (107.6°F)^40^. In our study, though in a few instances, the CWP and the insulating jacket failed to maintain the desired temperature range but did not go above 38.5⁰C at any point, therefore, using the thermal jacket will have no possibility of burning the neonate’s skin.

This study has several strengths. The number of events has enough power to capture the 95% success rate while adjusting for within CWPs correlations and other factors. The ambient temperature bands were randomly assigned to each of the events ensuring an equal number of reuses of the CWPs at each temperature band. This randomisation ensured an unbiased estimate of the effect of the ambient temperature on the outcomes. Moreover, to evaluate quality and safety of the insulating jacket fabrics, several tests such as pH test, formaldehyde test, azo dyes test, allergenic disperse dyes test, APEOs/ NPEOS test, and phthalates test were performed (Supplementary Table 7). Furthermore, we found handwashing with detergents are enough to clean the jacket, as evident in our virology report which suggests no existance of microorganism including *Klebsiella pneumoniae*, *E. coli* etc. (Supplementary Table 8). Moreover, to ensure more safety, we also have tested the alcohol wiping to clean the jacket and did not observe any negative reaction such as fabric fading, tearing, discoloration etc. However, suggested by the expert panel including members of virology, and neonatology, we opted out alcohol wiping since it is not recommended method of cleaning neonatal garments, instead we investigated whether autoclaving is suitable for the jacket and found autoclaving can be performed on the jackets without resulting any undesired condition. All the test results suggest the jacket fabrics are safe and have the highest quality to use for preterm and LBW neonates.

This study also has a few limitations. The temperature variation was obtained using controlled room temperature. We did not have any control over other ambient conditions such as humidity, air pressure etc. Therefore, the actual effect of seasonal variation couldn’t be captured due to resource and time constraints. Moreover, the survival distribution of the retention time for both the CWP and the insulating jacket could have been obtained if the testing process continued after 130 minutes. This would result in getting probabilities for greater retention times. Also, we used mannequin instead of animal model in this trial since it is not possible to find an appropriate animal model due to the difference of human newborn and animal newborn’s birth size, and gestational age.

## Conclusion

The development of the thermal jacket signifies a notable advancement in hypothermia management for preterm and LBW neonates. Our developed device demonstrated robust performance in preliminary testing; however, the next steps must center around real-world clinical evaluation and validations to substantiate our findings. As we proceed with additional research trials, the underlying goal remains clear: to contribute to the reduction of neonatal morbidity and mortality rates associated with hypothermia and create sustainable solutions that enhance the quality of care for the neonates born with prematurity or LBW. Therefore, further clinical testing among preterm or LBW neonates in health facility settings is needed to determine its safety, efficacy, and effectiveness for thermal care management.

## Electronic supplementary material

Below is the link to the electronic supplementary material.

Supplementary Material 1

## Data Availability

The study protocol and non-identifiable participants data will be shared upon reasonable request if the corresponding author’s institution approves sharing.
