# Supplementary material for: Testing of a reusable chemical warming pad and an insulating jacket to manage hypothermia of preterm or low birthweight neonates
Source: Sci Rep. 2025 Apr 10;15:12277. doi: 10.1038/s41598-025-96275-1 (PMC11985941; doi:10.1038/s41598-025-96275-1)
Supplement: Supplementary file 1 — Supplementary Material 1 [file 41598_2025_96275_MOESM1_ESM.doc]

**Testing of a reusable chemical warming pad and an insulating jacket to manage hypothermia of preterm or low birthweight neonates**

**Supplementary Table 1.** Test result of binary composites with Sodium Acetate Trihydrate (SAT)

| **Name of Additives^*^** | **Composition (g)** | **Peak temperature**  **at CWP**  **(in °C)** | **Retention time at CWP**  **(in min)** | **Results** |
| --- | --- | --- | --- | --- |
| **Graphite** | Aqueous solution of SAT + 0.5%  Graphite | 42 | 60 | Low retention with high temperature |
|  | Aqueous solution of SAT + 1.5%  Graphite | 44 | 65 |  |
| **Carboxyl Methyl**  **Cellulose (CMC)** | Aqueous solution of SAT + 1.5%  CMC | 45 | 90 | - Low retention with high temperature - Hardened after solidification |
|  | Aqueous solution of SAT + 10.0%  CMC | 47 | 120 |  |
| **Ethylene Glycol (EG)** | Aqueous solution of SAT + 10.0%  EG | 47 | 120 | Unstable |
|  | Aqueous solution of SAT + 15.0%  EG | 51 | 120 |  |
|  | Aqueous solution of SAT + 20.0%  EG | 48 | 120 |  |
| **Sodium Chloride**  **(NaCl)** | Aqueous solution of SAT + 2.0% | 45 | 76 | Low retention with high temperature |
|  | Aqueous solution of SAT + 0.5% | 42 | 70 |  |
| **Glycerol** | Aqueous solution of SAT + 5.0%  Glycerol | 43 | 100 | Low retention with high temperature |
|  | Aqueous solution of SAT + 15.0%  Glycerol | 42 | 90 |  |
|  | Aqueous solution of SAT + 5.0%  Paraffin | 52 | 100 | - Low retention with high temperature - Unstable |
| **Paraffin** | Aqueous solution of SAT + 0.5%  Paraffin | 45 | 90 |  |

^*^All the chemicals tested here are organic

**Supplementary Table 2.** Insulating jacket fabrics specification

| **Fabric** | **Biodegradable** | **Texture** | **Netted/ Woven** | **GSM** | **Purpose** |
| --- | --- | --- | --- | --- | --- |
| Single Jersey | Biodegradable | Plain Texture | Netted | 160 | For Baby comfort |
| Polyester-polar fleece | Non-Biodegradable | - | Netted | 200 | To retain heat of the jacket |
| Taffeta | Non-Biodegradable | - | Woven | 60 | To retain heat of the jacket |
| Screemline padding | Non-Biodegradable | - | Woven | - | To retain heat of the jacket |
| Air-permeable & water-resistant polyester | Non-Biodegradable | - | Woven | 82 | To resist water and air from outside to inside of the jacket |
| Polyester ripstop | Non-Biodegradable | Ripstic Texture | Woven | 100 | To resist water and pass all CWP heat to baby (heat conductivity High) |

**Supplementary Table 3.** Random assignment of temperature group for each chemical warming pad (CWP)

| **CWP ID** | **Number of times CWP used** | | | | | | | | |
| --- | --- | --- | --- | --- | --- | --- | --- | --- | --- |
|  | **1** | **2** | **3** | **4** | **5** | **6** | **7** | **8** | **9** |
| CWP 1 | 30-34°C | 25-29°C | 25-29°C | 18-24°C | 25-29°C | 30-34°C | 30-34°C | 18-24°C | 30-34°C |
| CWP 2 | 18-24°C | 18-24°C | 30-34°C | 25-29°C | 18-24°C | 25-29°C | 18-24°C | 30-34°C | 25-29°C |
| CWP 3 | 25-29°C | 30-34°C | 18-24°C | 30-34°C | 30-34°C | 18-24°C | 25-29°C | 25-29°C | 18-24°C |
| CWP 4 | 30-34°C | 25-29°C | 25-29°C | 18-24°C | 25-29°C | 30-34°C | 30-34°C | 18-24°C | 30-34°C |
| CWP 5 | 18-24°C | 18-24°C | 30-34°C | 25-29°C | 18-24°C | 25-29°C | 18-24°C | 30-34°C | 25-29°C |
| CWP 6 | 25-29°C | 30-34°C | 18-24°C | 30-34°C | 30-34°C | 18-24°C | 25-29°C | 25-29°C | 18-24°C |
| CWP 7 | 30-34°C | 25-29°C | 25-29°C | 18-24°C | 25-29°C | 30-34°C | 30-34°C | 18-24°C | 30-34°C |
| CWP 8 | 18-24°C | 18-24°C | 30-34°C | 25-29°C | 18-24°C | 25-29°C | 18-24°C | 30-34°C | 25-29°C |
| CWP 9 | 25-29°C | 30-34°C | 18-24°C | 30-34°C | 30-34°C | 18-24°C | 25-29°C | 25-29°C | 18-24°C |

**Supplementary Table 4.** Distribution of baseline and maximum temperature of CWP and insulating jacket over different factors

|  |  |  | **Baseline temperature (Mean)** | | | | **Maximum temperature (Mean)** | | | |
| --- | --- | --- | --- | --- | --- | --- | --- | --- | --- | --- |
|  |  |  | CWP | | Insulating jacket | | CWP | | Insulating jacket | |
| **Ambient temperature (°C)** | | | Mean | p-value^†^ | Mean | p-value^†^ | Mean | p-value^†^ | Mean | p-value^†^ |
| 18-24 | | | 31.13 | 0.19 | 29.83 | 0.02 | 37.86 | 0.26 | 37.79 | 0.16 |
| 25-29 | | | 31.95 |  | 31.58 |  | 37.82 |  | 37.62 |  |
| 30-34 | | | 31.43 |  | 31.09 |  | 37.7 |  | 37.67 |  |
| **Number of times CWP used** | | | |  |  |  |  |  |  |  |
| 1 | | | 31.66 | 0.97 | 29.68 | 0.18 | 37.92 | 0.92 | 37.90 | 0.74 |
| 2 | | | 31.83 |  | 29.31 |  | 37.84 |  | 37.72 |  |
| 3 | | | 31.67 |  | 31.01 |  | 37.70 |  | 37.63 |  |
| 4 | | | 31.34 |  | 30.90 |  | 37.78 |  | 37.65 |  |
| 5 | | | 31.37 |  | 32.71 |  | 37.84 |  | 37.69 |  |
| 6 | | | 31.36 |  | 30.72 |  | 37.83 |  | 37.69 |  |
| 7 | | | 30.87 |  | 30.64 |  | 37.80 |  | 37.68 |  |
| 8 | | | 31.52 |  | 31.36 |  | 37.68 |  | 37.60 |  |
| 9 | | | 31.92 |  | 31.14 |  | 37.73 |  | 37.66 |  |
| **Number of CWP** | | |  |  |  |  |  |  |  |  |
| 1 | | | 31.59 | 0.49 | 31.47 | 0.39 | 37.76 | 0.14 | 37.57 | 0.01 |
| 2 | | | 31.71 |  | 29.04 |  | 37.88 |  | 37.76 |  |
| 3 | | | 30.32 |  | 31.28 |  | 37.70 |  | 37.77 |  |
| 4 | | | 31.66 |  | 31.66 |  | 37.56 |  | 37.36 |  |
| 5 | | | 31.19 |  | 30.41 |  | 37.80 |  | 37.74 |  |
| 6 | | | 32.11 |  | 31.42 |  | 37.99 |  | 37.90 |  |
| 7 | | | 31.28 |  | 30.52 |  | 37.77 |  | 37.61 |  |
| 8 | | | 32.16 |  | 30.28 |  | 38.00 |  | 37.86 |  |
| 9 | | | 31.52 |  | 31.40 |  | 37.70 |  | 37.67 |  |

^†^p-value from analysis of variance

**Supplementary Table 5.** Distribution of time to reach temperature range between 36°C and 38°C for CWP and insulating jacket over different factors

| **Time to reach temperature between 36°C and 38°C (Minutes)** | | | | | | |
| --- | --- | --- | --- | --- | --- | --- |
|  | CWP | | | Insulating jacket | | |
|  | Mean | SD | p-value^†^ | Mean | SD | p-value^†^ |
| **Ambient temperature(°C)** |  |  |  |  |  |  |
| 18-24 | 0.84 | 0.40 |  | 1.12 | 0.60 |  |
| 25-29 | 0.93 | 0.71 | 0.40 | 1.10 | 0.79 | 0.53 |
| 30-34 | 1.07 | 0.71 |  | 1.32 | 0.98 |  |
| **Number of times CWP used** | | |  |  |  |  |
| 1 | 1.14 | 0.87 |  | 1.62 | 0.83 |  |
| 2 | 0.74 | 0.46 |  | 1.67 | 0.94 |  |
| 3 | 1.24 | 0.59 |  | 1.39 | 0.8 |  |
| 4 | 0.82 | 0.37 |  | 0.96 | 0.76 |  |
| 5 | 0.73 | 0.41 | 0.31 | 0.49 | 0.31 | 0.02 |
| 6 | 1.24 | 0.85 |  | 1.51 | 0.91 |  |
| 7 | 1.06 | 0.75 |  | 1.08 | 0.85 |  |
| 8 | 0.82 | 0.49 |  | 0.94 | 0.64 |  |
| 9 | 0.70 | 0.52 |  | 0.96 | 0.44 |  |
| **Number of CWP** |  |  |  |  |  |  |
| 1 | 1.70 | 0.48 |  | 1.70 | 0.67 |  |
| 2 | 0.91 | 0.61 |  | 1.36 | 0.94 |  |
| 3 | 0.71 | 0.33 |  | 0.69 | 0.45 |  |
| 4 | 1.30 | 0.71 |  | 1.38 | 0.71 |  |
| 5 | 0.88 | 0.37 | <0.001 | 1.04 | 0.47 | 0.07 |
| 6 | 0.57 | 0.34 |  | 1.19 | 1.34 |  |
| 7 | 0.93 | 0.36 |  | 1.31 | 0.50 |  |
| 8 | 0.99 | 1.01 |  | 1.36 | 0.92 |  |
| 9 | 0.52 | 0.25 |  | 0.59 | 0.36 |  |

^†^p-value from analysis of variance

**Supplementary Table 6.** Distribution of temperature (°C) and time (Minutes) for unsuccessful events

| **Temperature Band** |  | **No. of Events** | **First quartile** | **Second quartile** | **Third quartile** |
| --- | --- | --- | --- | --- | --- |
| **CWP** | **Temperature °C** | 6 | 38.10 | 38.26 | 38.50 |
|  | **Time** |  | 17 | 21 | 40 |
| **Insulating jacket** | **Temperature °C** | 2 | 38.10 | 38.40 | 38.58 |
|  | **Time** |  | 24.98 | 32.15 | 39.33 |

**Supplementary Table 7.** Quality and safety assessment tests of the insulating jacket fabrics

| **Tests** | **Justification for the tests** | **Results** |
| --- | --- | --- |
| pH test | To determine the value of pH and high pH value can create allergies and skin sensitivities | 6.3 (Neutral)  Not harmful for baby |
| Formaldehyde test | To identify the presence of this which can create allergies when it gets contact with skin | Negative |
| Azo dyes test | To ensure that there are no azo dyes that might cause cancer or skin sensitivity | Negative |
| Allergenic disperse dyes test | To detect the presence of dyes and It can create allergies and skin sensitivities | Negative |
| APEOs/NPEOS test | To identify the presence of moderately toxic metals and it may cause skin burns and eye irritation | Negative |
| Phthalates | To ensure the absence of carcinogenic substances which is very harmful when they suck it. | Negative |
| Migration of certain heavy metals | To ensure the absence of heavy metals that has negative effect on skin | Negative |
| Bisphenol test | To identify the presence of Bisphenol, its presence can negatively affect the early puberty, thyroid function and brain growth | Negative |

**Supplementary Table 8.** Safety test of the insulating jacket

| **Storage procedure** | **Quantity** | **Cleaning procedure** | **Gram-negative Enterobacteriaceae** | **Result** |
| --- | --- | --- | --- | --- |
| Insulating jacket stored in laboratory tested before 1^st^ time use | 2 | Detergent washed | - Klebsiella pneumoniae - E. coli - Acinetobacter baumannii | No growth  observed |
| Insulating jacket stored in laboratory  tested after being used on mannequin and washed | 2 | Detergent washed | - Klebsiella pneumoniae - E. coli - Acinetobacter baumannii | No growth  observed |
| Insulating jacket stored in laboratory tested after being used on mannequin | 1 | Autoclave | - Klebsiella pneumoniae - E. coli - Acinetobacter baumannii | No growth  observed |


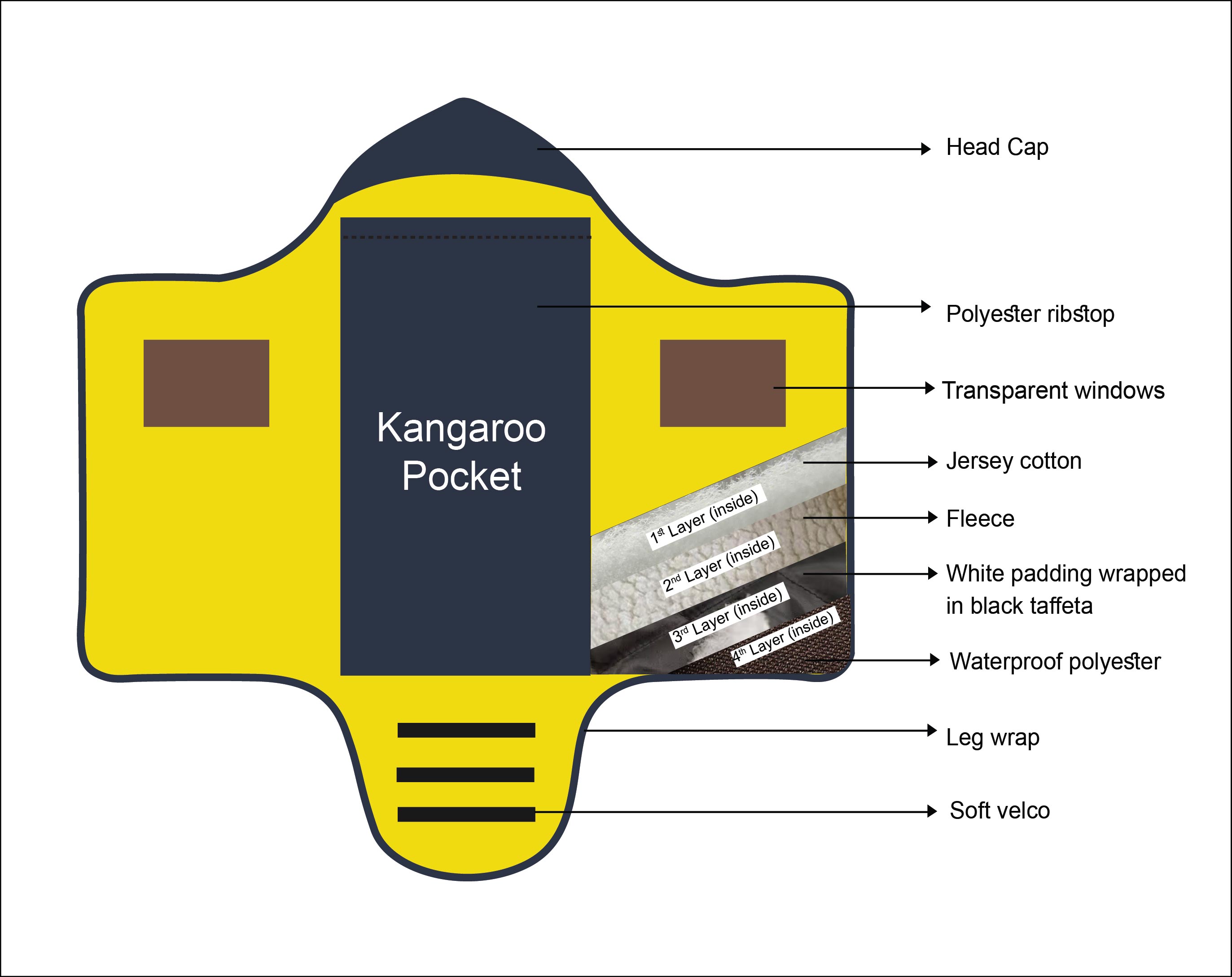


**Supplementary Figure 1.** Design of insulating jacket
